# Editorial: Targeting Developmental Pathways in Inflammation and Disease

**DOI:** 10.3389/fcell.2021.791115

**Published:** 2021-11-05

**Authors:** Eleni Anastasiadou, Lisa M. Minter, Maria Pia Felli

**Affiliations:** ^1^Department of Experimental Medicine, Sapienza University of Roma, Roma, Italy; ^2^Department of Veterinary and Animal Sciences, University of Massachusetts Amherst, Amherst, MA, United States

**Keywords:** developmental pathways, inflammatory responses, epigenetic modifications, tumor microenvironment, infectious agents

The collection provides an updated overview of the most relevant issues on how deregulation of signaling pathways impinges on different homeostatic and pathologic mechanisms, including birth defects and cancer. Indeed, in this issue of Frontiers in Cell and Developmental Biology many authors delineate how developmental pathways are intimately involved in inflammation and, with a high frequency, are fundamental mediators of virus-induced cancers.

Developmental pathways (DP) are critical molecular rheostats that drive a range of physiological processes, including embryonic development, lineage commitment, adult stem cell homeostasis, tissue regeneration, and immune response. Evolutionarily conserved signaling pathways, namely Sonic Hedgehog (SHH), Notch, and WNT, are frequently dysregulated in many cancer types. Indeed, genetic alterations of their members often result in excessive and sustained signals that drive transformation programs leading to the development of solid tumors and hematological malignancies. The same pathway, or even one member of it, can have a different biological outcome depending on the cell context, or can interact with inflammatory related pathways and further is able to modulate immune cell fate and function. Infectious agents, such as oncogenic viruses, cooperate with DP genes to impair the inflammatory response and evade immune surveillance. Notwithstanding many advances in DP research, there are still critical gaps in understanding various aspects in this field.

Our special issue encloses a group of papers with the intent to close these gaps and to highlight the intertwined relations between the major developmental pathways critically active in physiologic signaling networks or aberrantly functioning in developmental disorders or cancer (Figure 1).

**Figure 1:**
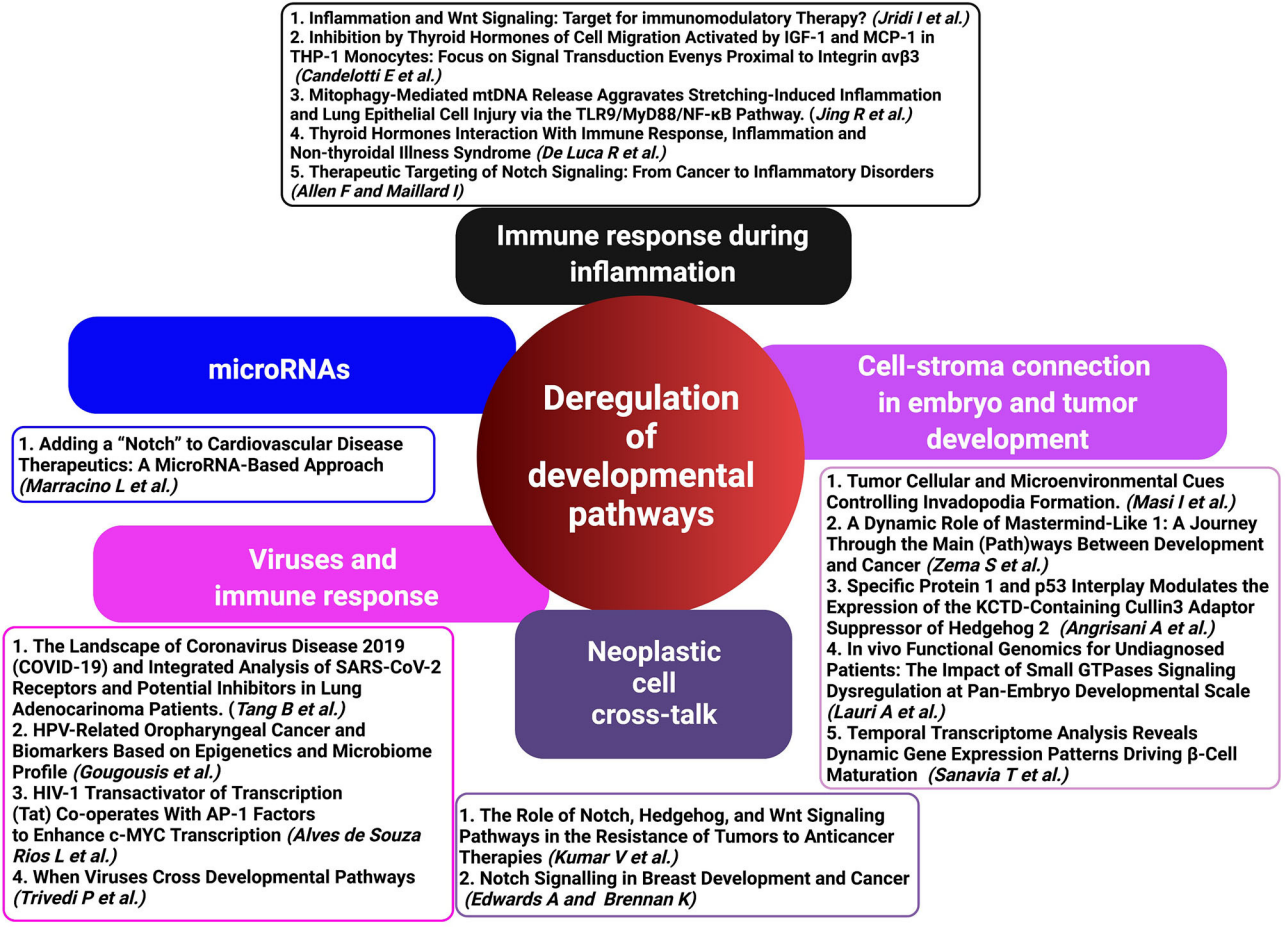
Deregulated developmental pathways (HH, Notch, and WNT) in inflammation, immune response, and cancer development represent critical crossroads for therapeutic intervention. A schematic representation of contributed articles to our special issue highlights the potential of targeting deregulated developmental pathways as a therapeutic approach in virus-associated diseases and malignancies (created with BioRender.com).

The review by Zema et al. offers a new perspective on the conventional role of Mastermind-like1 (MAML1) in the canonical Notch signaling pathway as well as how it functions unconventionally as a Notch-independent molecular switch that controls Hedgehog (HH), WNT/β-Catenin, and Hippo signaling pathways. MAML1 shows a strong ability to convert a plethora of stimuli in several biological processes during embryonic and post-natal life. Beyond this function, the versatile cofactor MAML1 can cooperate with different transcription factors in cell differentiation and cancer development.

Other key regulators of developmental signals and multiple cellular processes are monomeric GTPases of the RAS superfamily. The review by Lauri et al. comprehensively describes their dysregulation in developmental disorders and cancer. They further discuss the advantages of the zebrafish as an *in vivo* model and the latest molecular techniques available for investigating the dynamics of small GTPase-regulated processes during development and pediatric genetic diseases. Lastly, the authors stress the importance of the zebrafish model for enhancing our knowledge of rare diseases and for developing tailored therapies in precision medicine.

In embryonic morphogenesis, immune surveillance, as well as in cancer invasion and metastasis, cell motility is a tightly coordinated multistep process necessary for cells to reach their proper location. The review by Masi et al. provides an interesting description of the main signaling pathways involved in invadopodia formation during metastatic invasion, including growth factor receptors and integrins, as well as the activation of specific Rho GTPase family members. Signals received from the tumor microenvironment, such as metabolic conditions, mechanical signals, extracellular matrix, and interactions with stroma all affect invadopodia formation and activity. Many aspects and functions of invadopodia remain to be determined and, according to the authors, further studies are required to integrate the knowledge of how highly invasive cells sense and respond to the multitude of biochemical and biomechanical cues.

In their in-depth review, Edwards and Brennan provide insight into how the Notch signaling pathway participates both in normal mammary gland development as well as aberrant mechanisms that initiate or progress breast cancer. A prominent focus of this review is the crosstalk between components of the Notch pathway and other major signaling cascades, including the WNT pathway, to promote the hallmark characteristics of breast cancer cells such as proliferation, cell survival, invasion, and metastasis. Collaboration between canonical and non-canonical Notch signaling and other key cellular pathways can deregulate cell cycle progression and upregulate anti-apoptotic or pro-survival genes. The authors further discuss how Notch can promote epithelial-to-mesenchymal transitions especially through its intersection with microRNAs, and how it can confer therapeutic resistance in breast cancer stem cells. Finally, the authors provide a comprehensive table outlining the stages at which Notch signaling might be disrupted with targeted and/or combined therapies.

Resistance to anticancer therapeutics is a major barrier to long-lasting drug efficacy. Kumar et al. point to the crosstalk between Notch, HH, and WNT as a mechanism in chemo-resistance. Drug-induced up-regulation of the three pathways impinges on different cellular processes ranging from drug efflux to DNA damage response, or even to radiotherapy resistance. Moreover, promising cancer immunotherapy is also able to modulate immune cell functions in tumor microenvironment.

Toll-like receptors (TLRs) sense environmental danger signals and generate strong inflammatory immune responses. In their original article, Jing et al. interestingly associated mitophagy to mitochondrial (mt) DNA release which, when recognized by TLRs, activate the TLR9/myeloid differentiation factor 88 (MyD88)/nuclear factor-κB (NF-κB) signaling pathway, ultimately aggravating inflammation and lung injury. Targeting the MyD88/NF- κB pathway may represent a new therapeutic approach to preventing inflammation.

Two reviews elegantly dealt with the pathogenic function of Notch and WNT signaling pathways as modulators of immune cells in inflammation that can be therapeutically targeted.

Allen and Maillard outlined emerging concepts on the more recently investigated effects of Notch signaling in autoimmune and inflammatory disorders, essentially limited to preclinical disease models. Starting from Notch targeting in cancer and tumor angiogenesis they discuss successes and challenges encountered so far in Notch-based therapies in preclinical and clinical studies. From the lessons learned in cancer, they suggest that prolonged Notch inhibition is not the way to achieve long-lasting benefits in non-malignant inflammatory diseases. Instead, pulses of Notch inhibition at critical disease steps can reprogram immune cells to a less pathogenic state.

The concise and informative review by Jridi et al. focuses on how WNT signaling functions during normal and abnormal immune responses. The authors discuss the complex and, at times, contradictory ways WNT signaling regulates T cell activation and Th cell fate, including its regulation of NF-κB and the downstream biological functions it controls. Insightful discussions of WNT signaling in organ-specific and systemic inflammation disease models guides the reader through the complexities of WNT signaling in different organs. The authors conclude their review with a thoughtful discussion of how components of the WNT pathway might be targeted to provide therapeutic intervention in a number of disease conditions.

In their review, De Luca et al. discussed an interesting modulation of the WNT/β-catenin pathway by the thyroid hormone (TH) complex, T3–THRs–TREs, and how this interaction can influence various biological processes, including cell proliferation or homeostasis, as well as in cancer. For instance, in colorectal cancer the thyroid hormone receptor, THRα1, can activate β-catenin/Tcf4 transcription, which led to increased proliferation in the gut. The same review provided insight into how the T3–THRs–TREs complex increased miR-499 which inhibits calcineurin, a signaling molecule of Wnt/β-catenin pathway. This review and the original article by Candelotti et al. have opened up new avenues of research regarding interaction between thyroid hormones, developmental pathways, and inflammation. Interestingly, Sanavia et al., by using a multi-staged transcriptome analysis and protein-protein interaction network (PPI) approach, identified several genes involved in β-cell maturation. One of them was Hes1, a Notch target gene, which was significantly deregulated during this process, further confirming its role in pancreatic development.

In their original research article, Angrisani et al. investigate how the HH oncosuppressor, KCASH2, is regulated. Their findings suggest that there exists reciprocal regulation of KCASH2 by the transcription factor, Sp1, which is often overexpressed in tumors and the tumor suppressor p53. The authors identify putative binding sites on the *KCASH2* promoter for both proteins and offer evidence that Sp1 positively regulates KCASH2 while p53 acts as a negative regulator. When both proteins are present, it is the interplay between these two proteins that may ultimately determine KCASH2 levels. Paradoxically, in p53-deficient cells, the authors observed decreased KCASH2 levels, even in the presence of Sp1. This led them to further investigate dysregulated methylation mechanisms whereby they discovered upregulated DNA methyltransferase 1 (DNMT1) and decreased KCASH2. Inhibiting Sp1 or using a methyltransferase inhibitor could reverse this phenotype. In summary, the novel findings may pave the way for new therapeutic approaches to treating high HH-expressing tumors, that have lost p53 expression.

Finally, in the review by Marracino et al., the authors provide an in-depth discussion of the microRNAs that regulate Notch signaling, as well as those whose expression is Notch-dependent, in the context of cardiovascular disease (CVD). Covering such topics as arrhythmias, myocardial ischemia, and atherosclerosis, the authors summarize those instances where Notch signaling protects against or promotes CVD and speculate that targeting microRNAs acting up- or downstream of Notch signaling may represent a novel means of treating Notch-mediated CVD.

In our contribution to this special issue, we presented a review by Trivedi et al., that covered a vast area from the literature, on how viral proteins are able to dysregulate HH, Notch, or WNT signaling pathways and promote cancer. It is the first time that a review puts together an expansive amount of information regarding the function of different viral proteins which interact with the same genes in a particular pathway, such as Notch Intracellular domain (NICD) of the Notch pathway, to favor tumor progression. Furthermore, we have highlighted members of HH, Notch, and WNT pathways as possible common druggable targets for the treatment of virus-associated tumors as well as the link between immune evasion mechanisms and developmental pathways that viruses hijack to accelerate tumor growth. In addition, we brought many examples of viral protein interactions with microRNAs targeting DP genes that they can serve as novel therapeutic targets in cancer. Viral infections may result in the development of co-morbidities including cancer. Alves de Souza Rios et al. demonstrate that HIV-1 Tat enhances the expression of oncogenic c-MYC within the tumor cells of Burkitt lymphoma (BL) patients. The Tat/AP1 factor JunB protein complex up-regulates transcriptionally c-Myc promoter, that, as suggested by the authors, may contribute to the more aggressive phenotype observed in BL patients. In another article that focuses on viruses and cancer, Gougousis et al. explores the emerging role of HPV in the onset of oropharyngeal cancer (OC) and the use of mRNAs and microRNAs as biomarkers that can discriminate between the different stages of OC. As far as SARS-CoV-2 virus is concerned, Tang et al. performed a Gene Set Enrichment Analysis (GSEA) and identified the top 5 positive regulatory pathways in lung adenocarcinoma (LUAD) patients susceptible to SARS-CoV-2 infection, including the WNT signaling pathway. Furthermore, the authors identified miR-432-5p, which targets ACE2 and TMPRSS2 virus receptors, as well as AAK1, an endocytic regulator and upregulates the WNT pathway, as likely to favor tumor progression in the LUAD patients.

## Conclusions

Ongoing efforts to target a signaling system are a new therapeutic strategy in different types of diseases, from inflammation to cancer. It is our hope that this collection will serve to launch new studies with the aim to extend our understanding of DP crosstalk in the pathomechanisms of tumor development and progression, as well as in immune modulation during the inflammatory process. Our future challenge is to more fully understand how viruses co-opt different members of DP and microRNAs, which may open the way to more appropriate and safe combinatorial targeted therapies.

## Author Contributions

EA, LMM, and MF contributed equally to the article and approved it for publication. All authors contributed to the article and approved the submitted version.

## Conflict of Interest

The authors declare that the research was conducted in the absence of any commercial or financial relationships that could be construed as a potential conflict of interest.

## Publisher's Note

All claims expressed in this article are solely those of the authors and do not necessarily represent those of their affiliated organizations, or those of the publisher, the editors and the reviewers. Any product that may be evaluated in this article, or claim that may be made by its manufacturer, is not guaranteed or endorsed by the publisher.

